# *In Vitro* Generation of Brain Regulatory T Cells by Co-culturing With Astrocytes

**DOI:** 10.3389/fimmu.2022.960036

**Published:** 2022-07-15

**Authors:** Shinichi Yamamoto, Ako Matsui, Masaki Ohyagi, Chie Kikutake, Yoshihiro Harada, Mana Iizuka-Koga, Mikita Suyama, Akihiko Yoshimura, Minako Ito

**Affiliations:** ^1^ Department of Microbiology and Immunology, Keio University School of Medicine, Tokyo, Japan; ^2^ Division of Allergy and Immunology, Medical Institute of Bioregulation, Kyushu University, Fukuoka, Japan; ^3^ Division of Bioinformatics, Medical Institute of Bioregulation, Kyushu University, Fukuoka, Japan

**Keywords:** astrocyte, IL-33, Parkinson’s disease, serotonin, tissue Tregs

## Abstract

Regulatory T cells (Tregs) are normally born in the thymus and activated in secondary lymphoid tissues to suppress immune responses in the lymph node and at sites of inflammation. Tregs are also resident in various tissues or accumulate in damaged tissues, which are now called tissue Tregs, and contribute to homeostasis and tissue repair by interacting with non-immune cells. We have shown that Tregs accumulate in the brain during the chronic phase in a mouse cerebral infarction model, and these Tregs acquire the characteristic properties of brain Tregs and contribute to the recovery of neurological damage by interacting with astrocytes. However, the mechanism of tissue Treg development is not fully understood. We developed a culture method that confers brain Treg characteristics *in vitro*. Naive Tregs from the spleen were activated and efficiently amplified by T-cell receptor (TCR) stimulation in the presence of primary astrocytes. Furthermore, adding IL-33 and serotonin could confer part of the properties of brain Tregs, such as ST2, peroxisome proliferator-activated receptor γ (PPARγ), and serotonin receptor 7 (Htr7) expression. Transcriptome analysis revealed that *in vitro* generated brain Treg-like Tregs (induced brain Tregs; iB-Tregs) showed similar gene expression patterns as those in *in vivo* brain Tregs, although they were not identical. Furthermore, in Parkinson’s disease models, in which T cells have been shown to be involved in disease progression, iB-Tregs infiltrated into the brain more readily and ameliorated pathological symptoms more effectively than splenic Tregs. These data indicate that iB-Tregs contribute to our understanding of brain Treg development and could also be therapeutic for inflammatory brain diseases.

## Introduction

Regulatory T cells (Tregs) normally account for about 5%–10% of CD4^+^ T cells and are present in the lymphoid tissues and at inflammation sites. However, Tregs have recently been shown to accumulate in a steady or inducible manner in a variety of non-lymphoid tissues, including fat, muscle, skin, lung, intestine and brain (1). These tissue Tregs recognize self-antigen characteristic of tissues and have a limited T-cell receptor (TCR) repertoire. Tissue Treg gene expression analysis in various organs has uncovered a phenotype that is different from that of lymphoid tissue depending on the organ (1–3). Common features of tissue Tregs include higher expression of genes such as *Il10, Il1rl1* (encoding the IL-33 receptor ST2), *Pparg, Areg* (amphiregulin, a type of ligand for the epidermal growth factor [EGF] receptor), and *Klrg1, Ctla4, Tigit, Gata3, Batf*, and *Irf4* as well as lower expression of *Lef1, Tcf7*, and *Bcl2* compared with lymphoid Tregs (2).

The localized microenvironment may play an important role in the tissue-specific Treg phenotypes. Single-cell RNA sequencing (scRNAseq) technology has revealed that tissue Tregs gradually acquire their properties in the associated lymph nodes and become mature tissue Tregs within tissues (4, 5). Analysis of transgenic mice with TCRs that are highly abundant in adipose Tregs showed that adipose Tregs at the priming phase in lymphoid tissues express low levels of peroxisome proliferator-activated receptor γ (PPARγ) infiltrate into adipose tissues to become the final mature adipose Tregs that express high levels of PPARγ (6).

Skeletal muscle Tregs have also been well studied, and Areg has been shown to be a Treg-derived repair factor for muscle damage (7). Tregs in muscle tissue express high levels of the IL-33 receptor, ST2, and IL-33 has been shown to promote muscle Treg accumulation (8). In the kidney, CCR4^+^GATA3^+^Tregs accumulate during the chronic phase of renal injury and promote recovery of renal function by suppressing Th1 in the injured region induced by basement membrane antibodies (9). Furthermore, PPARγ agonists increase renal Treg levels and accelerate recovery from renal injury (9).

Using an experimental cerebral ischemia–reperfusion model (middle cerebral artery occlusion; MCAO) for stroke, we and others have shown that Tregs accumulated during the chronic phase, 2 weeks after disease onset (10, 11). Brain Tregs, similar to other tissue Tregs, are Helios^+^ tTregs with a special TCR repertoire and high CTLA-4, PD-1, Areg, and ST2 expression. Similar to other tissue Tregs, TCR signaling, IL-2, and IL-33 are essential for proliferation in the brain. Additionally, brain Tregs also express several neural-related genes, particularly serotonin receptor 7 (Htr7), which increases cAMP levels. Serotonin stimulated proliferation of brain Tregs and improved neurological symptoms *via* Htr7. As a mechanism of recovery from neurological symptoms, brain Tregs suppress excessive astrocyte activation, which is called astrogliosis, by secreting Areg (10). Another recent study showed that Treg cell-derived osteopontin acts *via* integrin receptors in microglia to enhance microglial repair activity, which in turn promotes oligodendrocyte neogenesis and white matter repair (11). Increasing the number of Treg cells by administering IL-2:IL-2 antibody complexes after stroke improved the white matter status and restored neurological function in the long-term, suggesting that Tregs could be a therapeutic target of neural recovery after stroke (11).

In neurodegenerative diseases such as Parkinson’s disease, antigen-specific Tregs have also been reported to interact with glial cells, thereby suppressing neuroinflammation and promoting neuronal survival (12). A vasoactive intestinal peptide agonist has been shown to increase Treg activity thereby suppressing inflammatory microglia and increasing survival of dopaminergic neurons (13).

Although studies of tissue Tregs have made remarkable progress in recent years, the molecular mechanisms of their development remain unclear. Molecules that facilitate tissue Treg development and specific tissue antigens are largely unknown. In this study, we attempted to generate brain Tregs *in vitro* to elucidate the induction mechanism of brain Tregs. We found that co-culturing astrocytes and adding several cytokines and bioactive substances facilitated brain Treg-like differentiation. Furthermore, brain Treg-like cells obtained by this method efficiently infiltrated and suppressed inflammation in the brain. This study provides the basis for elucidating the developmental mechanism of tissue Tregs and their clinical application.

## Results

### Treg Expansion and Activation by Co-Culturing with Primary Astrocytes

First, we hypothesized that Treg activation and proliferation are required for tissue Treg development, because tissue Tregs are thought to be related to effector Tregs (14). When Tregs from mouse spleen were cultured *in vitro* in the presence of IL-2 and anti-CD3/CD28 antibodies, Tregs did not proliferate well and majority of them died in culture within 3 to 6 days (data not shown). Since brain Tregs are expected to interact with tissue nonimmune glial cells, we first co-cultured splenic Tregs with slices of brain sections. However, Tregs did not proliferate (data not shown). Therefore, we co-cultured splenic Tregs with primary brain cells, astrocytes, and microglia. Primary astrocytes and microglia were obtained from neonatal mouse brain as reported (15–17).

**Figure 1:**
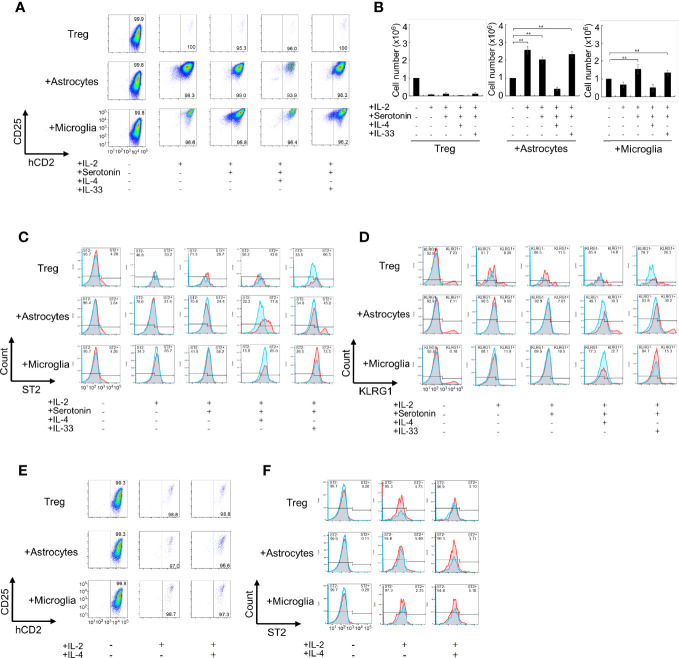
Co-culture of Tregs with primary astrocytes and microglia *in vitro.* **(A–D)** Tregs alone or co-cultured with astrocytes or microglia in the presence of indicated cytokines (20 ng/mL) or serotonin (10 μM) in the presence of IL-2 and anti-CD3/CD28 antibodies for 6 days. The Foxp3 and CD25 expression was analyzed using FACS **(A)**, Foxp3^+^Treg cell number was estimated **(B)**, and ST2 and KLRG1 expression was analyzed using FACS **(C, D)**. The left panels [all cytokines and serotonin are (-)] are shown as before co-culture. **(E, F)** Effect of transwell on the activation **(E)** and ST2 expression **(F)** of Treg under co-culture with astrocytes. In **(C, D, F)**, Blue fines were without first antibody, Red lines were with first antibody, and overlapping regions were shown in gray. * P<0.05 ** P<0.01.

We evaluated the activation stage of Tregs using CD25, Foxp3, and ST2 (a marker of tissue Tregs) expression as well as proliferation. We found that co-culture with microglia or with both astrocytes and microglia resulted in Treg activation but low proliferation, whereas co-culture with astrocytes alone resulted in proliferation as well as strong Treg activation. Next, we added cytokines and bioactive substances to the Tregs and astrocyte co-culture. Significant upregulation of CD25 and Foxp3 expression was observed when IL-2, IL-4, IL-33, brain-derived neurotrophic factor (BDNF), Areg, and serotonin were added to the co-culture system (**Figures 1A, B**, and **Table 1**). Among factors we examined, IL-4 most strongly upregulated ST2 expression but had a negative effect on cell proliferation. Conversely, IL-33 and serotonin had a positive effect on proliferation but these factors alone showed little effects on ST2 expression (**Table 1**). However, combination of IL-33 and serotonin upregulated ST2 expression (**Figure 1C**).

**Table 1:** The effect of various factors on Treg activation and proliferation.

	Astrocytes			Microglia		
Reagents	Activation	Proliferation	Protein	Activation	Proliferation	Protein
	(CD25^high^ hCD2^high^)	(Cell number)	(ST2)	(CD25^high^ hCD2^high^)	(Cell number)	(ST2)
IL-2 (20 ng/ml)	**++**	**-**	**-**	**+**	**-**	**-**
IL-1β (20 ng/ml)	**+**	**-**	**-**	**+**	**-**	**-**
1L-4 (20 ng/ml)	**++**	**-**	**+**	**+**	**-**	**-**
1L-6 (20 ng/ml)	**+**	**-**	**-**	**+**	**-**	**-**
IL-10 (20 ng/ml)	**+**	**-**	**-**	**+**	**-**	**-**
IL-33 (20 ng/ml)	**++**	**+**	**-**	**+**	**+**	**+**
TNFα (20 ng/ml)	**+**	**-**	**-**	**+**	**-**	**-**
EGF (20 ng/ml)	**+**	**-**	**-**	**+**	**-**	**-**
TSLP (20 ng/ml)	**+**	**-**	**-**	**+**	**-**	**-**
M-CSF (20 ng/ml)	**+**	**-**	**-**	**+**	**-**	**-**
BDNF (20 ng/ml)	**++**	**-**	**-**	**+**	**-**	**-**
Amphiregulin (20 ng/ml)	**++**	**-**	**-**	**+**	**-**	**-**
PGE2 (IpM)	**+**	**-**	**-**	**-**	**-**	**-**
Pioglitazone (1 μM)	**+**	**-**	**-**	**+**	**-**	**-**
Serotonin (10 μM)	**++**	**+**	**-**	**+**	**+**	**-**
LPS (500 ng/ml )	**+**	**-**	**-**	**+**	**-**	**-**
Vitamin A (25 nM)	**+**	**-**	**-**	**+**	**-**	**-**
Vitamin C (10 μg/ml)	**+**	**-**	**-**	**+**	**-**	**-**

Splenic Tregs were co-cultured with astrocytes or microglia for 3 days in the presence of the indicated factors and then examined for Treg activation (Foxp3^high^ and CD25 ^high^ expression), proliferation, and ST2 expression.

-, no expression; +, positive; ++, highly positive (see FACS data in **Figure 1**). hCD2 means Foxp3.

Next, we performed co-cultures in the presence of combined factors that promoted Treg activation and proliferation. Among combinations of factors listed in **Table 2** in co-culture with astrocytes, the combination of IL-2, IL-33, and serotonin most efficiently promoted proliferation and induced ST2, KLRG1, and *Htr7* expression, which are characteristic of brain Tregs (**Figures 1A–D**, and **Table 2**). While no induction was observed in the co-culture with microglia. Only IL-4 induced Areg expression, but IL-33 and serotonin did not have this effect (**Table 2**). All these data suggest that IL-2 and TCR signals were sufficient for CD25 and Foxp3 expression and proliferation, however, IL-4 or IL-33 and astrocytes were required for ST2, KLRG1, and *Htr7* expression. Although PPARγ agonists were shown to increase tissue Tregs *in vivo*, we did not observe any strong effects *in vitro* (**Table 2**).

**Table 2:** The effect of cytokine and factor combinations on Treg activation, proliferation, and expression of brain Treg markers.

Cells		Astrocytes							Microglia		
Reagents	IL-2 (20 ng/ml)	**〇**	**〇**	**〇**	**〇**	**〇**	**〇**	**〇**	**〇**	**〇**	**〇**
	IL-4 (20 ng/ml)	**〇**		**〇**		**〇**			**〇**		
	IL-33 (20 ng/ml)	**〇**	**〇**	**〇**	**〇**	**〇**	**〇**	**〇**	**〇**	**〇**	**〇**
	Serotonin (10 μM)	**〇**	**〇**	**〇**	**〇**	**〇**	**〇**	**〇**	**〇**	**〇**	**〇**
	BDNF (20 ng/ml)	**〇**	**〇**								
	Amphiregulin (20 ng/ml)	**〇**	**〇**	**〇**	**〇**						
	Pioglitazone (1 μg/ml)	**〇**	**〇**	**〇**							
	CCL20 (20 ng/ml)							**〇**			**〇**
	CCL1(20 ng/ml)							**〇**			**〇**
Proteins	ST2	**+**	***-***	**+**	**+**	**-**	**+**	**+**	**-**	**-**	**-**
	KLRG1	**-**	**-**	**-**	**+**	**-**	**+**	**+**	**-**	**-**	**-**
mRNA	*Areg*	**+**	**-**	**++**	**-**	**-**	**-**	**-**	**-**	**-**	**-**
	*Htr7*	**+**	**++**	**+**	**+**	**-**	**++**	**+**	**-**	**++**	**+**
Activation (CD25^high^ hCD2^high^)		**+**	**+**	**+**	**+**	**-**	**+**	**+**	**-**	**+**	**+**
Proliferation		**-**	**+**	**-**	**-**	**-**	**+**	**+**	**-**	**+**	**+**

Splenic Tregs were co-cultured with astrocytes or microglia for 6 days in the presence of factor combinations (o). Brain Treg markers ST2 and KLRG1 were analyzed using FACS, and Areg and Htr7 were analyzed using qPCR. Treg activation (Foxp3^high^ and CD25 ^high^ expression) and proliferation were also analyzed. -, no increase; +, positive; ++, highly positive.

Lastly, we performed more detailed analyses of these combinations (**Table 3**). IL-4 upregulated ST2 and Areg expression, but we excluded it because IL-4 suppressed Treg cell proliferation and did not yield the required number of cells for the experiment. IL-33 and serotonin combination induced brain Treg-like cells, as determined by ST2 and *Htr7* expression. However, adding IL-33 and serotonin did not strongly induce *Areg*, *Ccr6, and Il10* (**Table 3**). Co-culture with microglia or microglia plus astrocytes or slices of brain sections did not induce markers such as *Gata3*, ST2, or KLRG1 (**Table 3**). We concluded that co-culture of splenic Tregs with primary astrocytes in the presence of anti-CD3/CD28 antibodies, IL-2, IL-33 and serotonin is the most preferable condition to induce brain Treg-like characters *in vitro*.

**Table 3:** Expression of brain Treg markers by the indicated combination of cytokines.

Ceils		Treg				Astrocytes				Microglia				Astrocytes Microglia				Brain sections			
	IL-2 (20 ng/ml)	**〇**	**〇**	**〇**	**〇**	**〇**	**〇**	**〇**	**〇**	**〇**	**〇**	**〇**	**〇**	**〇**	**〇**	**〇**	**〇**	**〇**	**〇**	**〇**	**〇**
Reagents	Serotonin (10 μM*)*		**〇**	**〇**	**〇**		**〇**	**〇**	**〇**		**〇**	**〇**	**〇**		**〇**	**〇**	**〇**		**〇**	**〇**	**〇**
	IL-4 (20 ng/ml)			**〇**				**〇**				**〇**				**〇**				**〇**	
	IL-33 (20 ng/ml)				**〇**				**〇**				**〇**				**〇**				**〇**
Activation (CD25^high^ hCD2^high^)		**-**	**-**	**-**	**-**	**+**	**+**	**+**	**+**	**+**	**+**	**+**	**+**	**+**	**+**	**+**	**+**	**+**	**+**	**+**	**+**
Proliferation		**-**	**-**	**-**	**-**	**+**	**+**	**-**	**+**	**-**	**+**	**-**	**+**	**-**	**-**	**-**	**-**	**-**	**-**	**-**	**-**
Proteins	ST2	**-**	**-**	**+**	**-**	**-**	**-**	**+**	**+**	**-**	**-**	**-**	**-**	**-**	**-**	**+**	**-**	**-**	**-**	**-**	**-**
	KLRG1	**+**	**+**	**+**	**+**	**-**	**-**	**+**	**+**	**-**	**-**	**-**	**-**	**-**	**-**	**-**	**-**	**-**	**-**	**-**	**-**
	CCR6	**-**	**-**	**-**	**-**	**-**	**-**	**-**	**-**	**-**	**-**	**-**	**-**	**-**	**-**	**-**	**-**	**-**	**-**	**-**	**-**
	CCR8	**-**	**-**	**-**	**-**	**+**	**+**	**+**	**+**	**+**	**+**	**+**	**+**	**+**	**+**	**+**	**+**	**+**	**+**	**+**	**+**
mRNA	*Areg*	**-**	**-**	**-**	**-**	**-**	**-**	**-**	**-**	**-**	**-**	**-**	**-**	**-**	**-**	**-**	**-**	**-**	**-**	**-**	**-**
	*Htr7*	**-**	**-**	**-**	**-**	**+**	**+**	**-**	**++**	**-**	**+**	**-**	**++**	**-**	**-**	**-**	**-**	**-**	**-**	**-**	**-**
	*Gata3*	**-**	**-**	**+**	**-**	**+**	**-**	**-**	**++**	**-**	**-**	**+**	**-**	**-**	**-**	**-**	**-**	**-**	**-**	**-**	**-**
	*Pparg*	**-**	**-**	**-**	**-**	**-**	**+**	**++**	**+**	**-**	**-**	**+**	**-**	**+**	**+**	**+**	**+**	**+**	**+**	**+**	**++**
	*Il1rl1*	**-**	**-**	**+**	**-**	**-**	**-**	**+**	**++**	**-**	**-**	**-**	**-**	**-**	**-**	**+**	**-**	**-**	**-**	**-**	**-**
	*Klrg1*	**+**	**+**	**++**	**+**	**-**	**-**	**+**	**++**	**-**	**-**	**-**	**-**	**-**	**-**	**-**	**-**	**-**	**-**	**-**	**-**
	*Ccr6*	**-**	**-**	**-**	**-**	**-**	**-**	**-**	**-**	**-**	**-**	**-**	**-**	**-**	**-**	**-**	**-**	**-**	**-**	**-**	**-**
	*Ccr8*	**-**	**-**	**-**	**-**	**+**	**-**	**-**	**++**	**-**	**-**	**+**	**++**	**-**	**-**	**-**	**-**	**-**	**-**	**-**	**-**
	*Il10*	**-**	**-**	**-**	**-**	**-**	**-**	**-**	**-**	**-**	**-**	**-**	**-**	**-**	**-**	**-**	**-**	**-**	**-**	**-**	**-**
	*Ednrb*	**-**	**-**	**-**	**-**	**+**	**+**	**+**	**++**	**+**	**+**	**+**	**++**	**+**	**+**	**+**	**+**	**++**	**+**	**+**	**+**

Splenic Tregs were co-cultured with astrocytes, microglia, or both or with brain sections in the presence of indicated factors (o) for 6 days. Treg activation (Foxp3^high^ and CD25 ^high^ expression), proliferation, and protein expression of ST2, KLRG1, CCR6, and CCR8 were determined using FACS and Areg and other brain Treg marker genes were measured using qPCR, -, no increase; +, positive; ++, highly positive.

### Tregs Co-Cultured With Astrocytes in the Presence of IL-33 and Serotonin Can Generate Brain Treg-Like Cells *In vitro*

Next, we examined whether Tregs induced brain Treg-like Tregs in a co-culture that included these factors with astrocytes in the absence of or in contact with astrocytes. First, we examined Treg activation and ST2 expression when Tregs and astrocytes or microglia were separated using a transwell during co-culture (**Figures 1E, F**). Treg activation and ST2 expression were not observed without direct contact between Tregs and astrocytes. When the astrocyte or microglia culture supernatant (CM, conditioned medium; ACM, CM from astrocytes; and MCM, CM from microglia) was used instead of an astrocyte/microglia cell layer, but proliferation, activation, and induction of ST2 in Tregs were not observed (**Supplementary Figures 1A, B**). In co-cultures with astrocytes, adding additional MCM did not further increase CD25, Foxp3, and ST2 (**Supplementary Figures 1C, D**). These results indicate that contact with astrocytes is necessary for Tregs to be induced into brain Treg-like property *in vitro*.

### Characterization of *In Vitro* Generated Brain Treg-Like Cells

**Figure 2:**
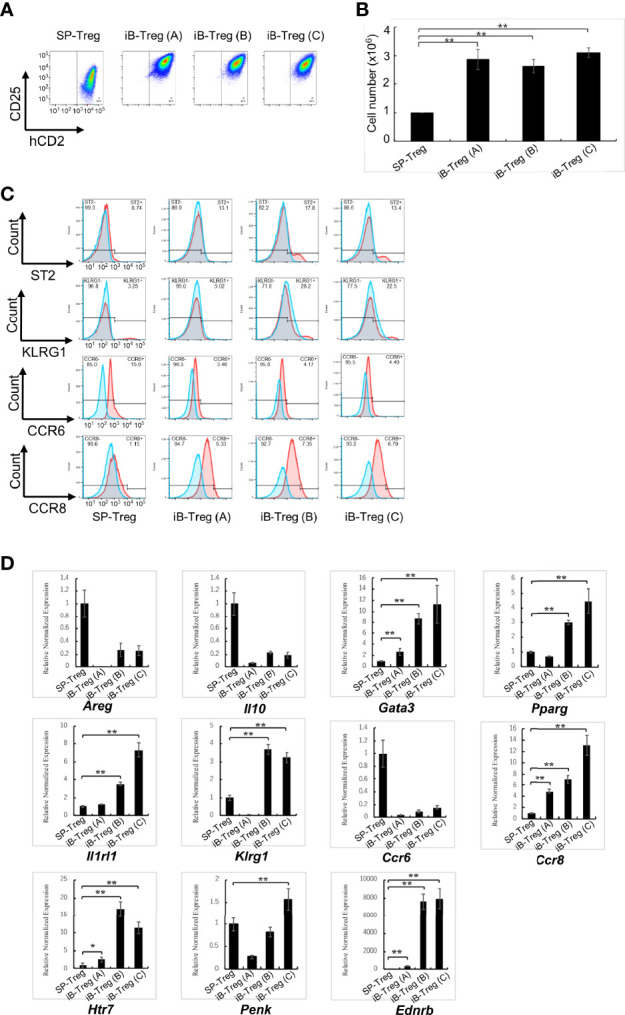
Characterization of *in vitro*-generated iB-Tregs. SP-Tregs were co-cultured i) with astrocytes in the presence of IL-2 and anti-CD3/CD28 antibodies [iB-Treg (A)]; ii) with astrocytes in the presence of IL-2, IL-33, serotonin and anti-CD3/CD28 antibodies [iB-Treg (B)]; or iii) with astrocytes in the presence of IL-2, IL-33, serotonin, CCL1, CCL20, and anti-CD3/CD28 antibodies [iB-Treg (C)] for 6 days. Blue fines were without first antibody, Red lines were with first antibody, and overlapping regions were shown in gray **(C)**. Then CD25/Foxp3 (hCD2) expression **(A)** was analyzed using FACS and the number of cells after co-culture was determined **(B)**. Expression of the indicated tissue Treg markers were analyzed using FACS **(C)** and RT-qPCR **(D)**. * P<0.05 ** P<0.01.

On the basis of the above results, we further characterized *in vitro* generated brain Treg-like cells (iB-Treg cells), which were induced through co-culture with astrocytes under several conditions; only in the presence of IL-2 and anti-CD3/28 antibodies (iB-Tregs (A)); co-culture with astrocytes in the presence of IL-2, IL-33, serotonin, and anti-CD3/28 antibodies (iB-Treg (B)); or co-culture with astrocytes in the presence of IL-2, IL-33, serotonin, and anti-CD3/28 antibodies as well as the CCR8-ligand CCL1 and CCR6-ligand CCL20 (iB-Treg (C)). Chemokines, CCL1 and CCL20 were included with an expected increase in their receptors, CCR6 and CCR8. Treg activation (CD25 and Foxp3 expression) and proliferation were markedly observed in all culture systems (**Figures 2A, B**). Among brain Treg markers, ST2, KLRG1, and CCR8 were expressed in iB-Tregs (B) and iB-Tregs (C), while CCR6 was decreased compared with splenic Tregs. Adding CCL1 and CCL20 had no significant effect on the properties of iB-Tregs (**Figure 2C**). Gene expression analysis revealed that *Gata3, Pparg, Il1rl1, Klrg1, Ccr8, Htr7, Penk* (Proenkeohalin gene), and *Ednrb* (Endothelin receptor type B gene) expression levels were elevated in iB-Tregs (B) and (C) (**Figure 2D**). Therefore, adding CCL1 and CCL20 had no synergistic effect on the properties of iB-Tregs.

**Figure 3:**
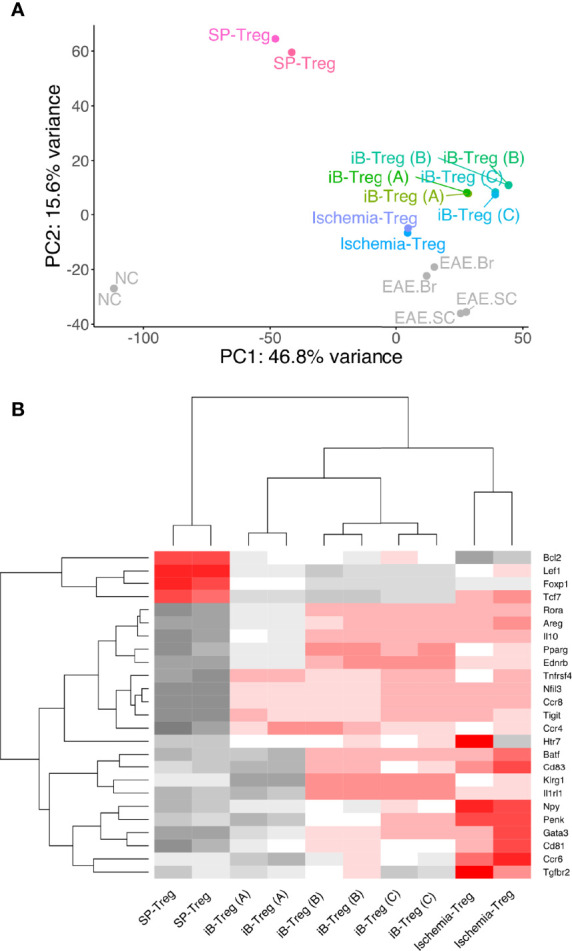
Comparison of gene expression between brain Tregs and iB-Tregs. The three iB-Treg **(A–C)** conditions were described in **Figure 2**. Total RNA of Tregs from spleen, ischemic brain, EAE brain or EAE spinal cord (SC), or iB-Tregs after 6 days of co-culture was isolated, and sequenced. **(A)** PC analysis (SP-Treg, Ischemia-Treg, iB-Treg (A), iB-Treg (B), iB-Treg (C), NC, EAE.Br and EAE.SC). **(B)** heatmap analysis [SP-Treg, Ischemia-Treg, iB-Treg (A), iB-Treg (B) and iB-Treg (C)] for representative brain Treg genes.

To compare the characteristics of iB-Tregs with *in vivo* brain Tregs, total RNA-seq analysis and principal component analysis (PCA) of Tregs were performed (**Figure 3A**). We confirmed that iB-Tregs are different from splenic Tregs and are similar to brain Tregs that are isolated from brains of mice with cerebral infarction or experimental autoimmune encephalomyelitis (EAE) (**Figure 3A**). Similar to quantitative polymerase chain reaction (qPCR) results shown in **Figure 2D**, heat map analysis revealed that individual brain Treg markers such as *Gata3, Pparg, Il1rl1, Klrg1, Penk*, and *Htr7* were more highly expressed in iB-Tregs (B) and (C) than in spleen Tregs, which is similar to that of ischemic brain Tregs (**Figures 3A, B**). These data indicate that iB-Tregs share many of the brain Treg properties, even though they are not completely identical to brain Tregs.

### iB-Tregs Preferentially Migrate Into the Brain and Ameliorate Symptoms in a Parkinson’s Disease Model

**Figure 4:**
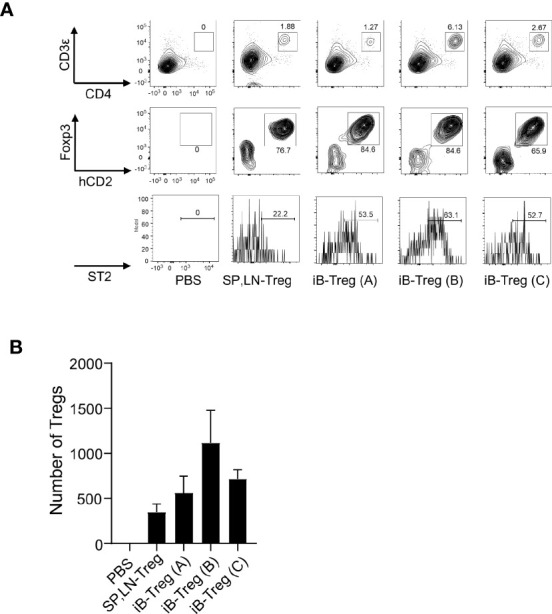
Infiltration of iB-Tregs into the injured brain. CD3-deficient mice with ischemic brain injury were generated using the MCAO model, as described. Splenic and lymph node Tregs (SP, LN-Treg) or iB-Tregs **(A–C)** after 6 days of co-culture (5 × 10^5^ cells/mice N=3-4) were transferred into the CD3-deficient mice on day 5 after stroke onset. Seven days later, ST2 expression **(A)** and cell number **(B)** of Foxp3^+^Tregs in the brain were analyzed using FACS. Data are presented as the average of three independent experiments.

We then examined the effects of iB-Tregs after *in vivo* transfer. First, we examined the infiltration of Tregs into the brain of mice using a cerebral infarction model. After transferring iB-Tregs (A, B, and C) into T cell-deficient mice with induced cerebral infarction, we measured the number of Tregs infiltrating into the brain and the ST2 expression level. As shown in **Figure 4A**, iB-Tregs, especially B-type iB-Tregs, tended to infiltrate the injured brain more efficiently than splenic Tregs and showed highest ST2 expression. Although the differences in the number of iB-Tregs were not statistically significant, we confirmed that iB-Tregs, especially, iB-Treg (B), tended to accumulate more efficiently than splenic Tregs (**Figure 4B**).

In EAE, Treg transfer has been shown to improve clinical scores (18). As expected, B- and C-type iB-Tregs had a greater therapeutic effect than splenic Treg and A-type iB-Treg (**Supplementary Figures 2A, B**). Unfortunately, the number of cells that infiltrated the brain was too small to judge whether B- and C-type iB-Tregs infiltrate into the brain more efficiently than splenic Tregs or A-type iB-Tregs. This may be because T cells in the spinal cord are more important for EAE symptoms than those in the brain (19).

Next, we used a Parkinson’s disease model in which the neurotoxin 1-methyl-4-phenyl-1,2,3,6-tetrahydropyridine (MPTP) is administered to cause degeneration of dopaminergic neurons. T cells, especially Th17 cell, have been reported to be involved in neurodegeneration, and Tregs inhibit symptoms of the disease model (20–22). As previously reported, T-cell removal by X-ray irradiation followed by MPTP administration reduced T-cell infiltration into the brain and suppressed the decrease of tyrosine hydroxylase (TH)-positive dopaminergic neurons in the substantia nigra, the increase in the number of α-synuclein phosphorylation, and the increase in microglial activation (Iba1^+^ cells), all of which are indicators of neurodegeneration (**Supplementary Figures 3A–G**).

**Figure 5:**
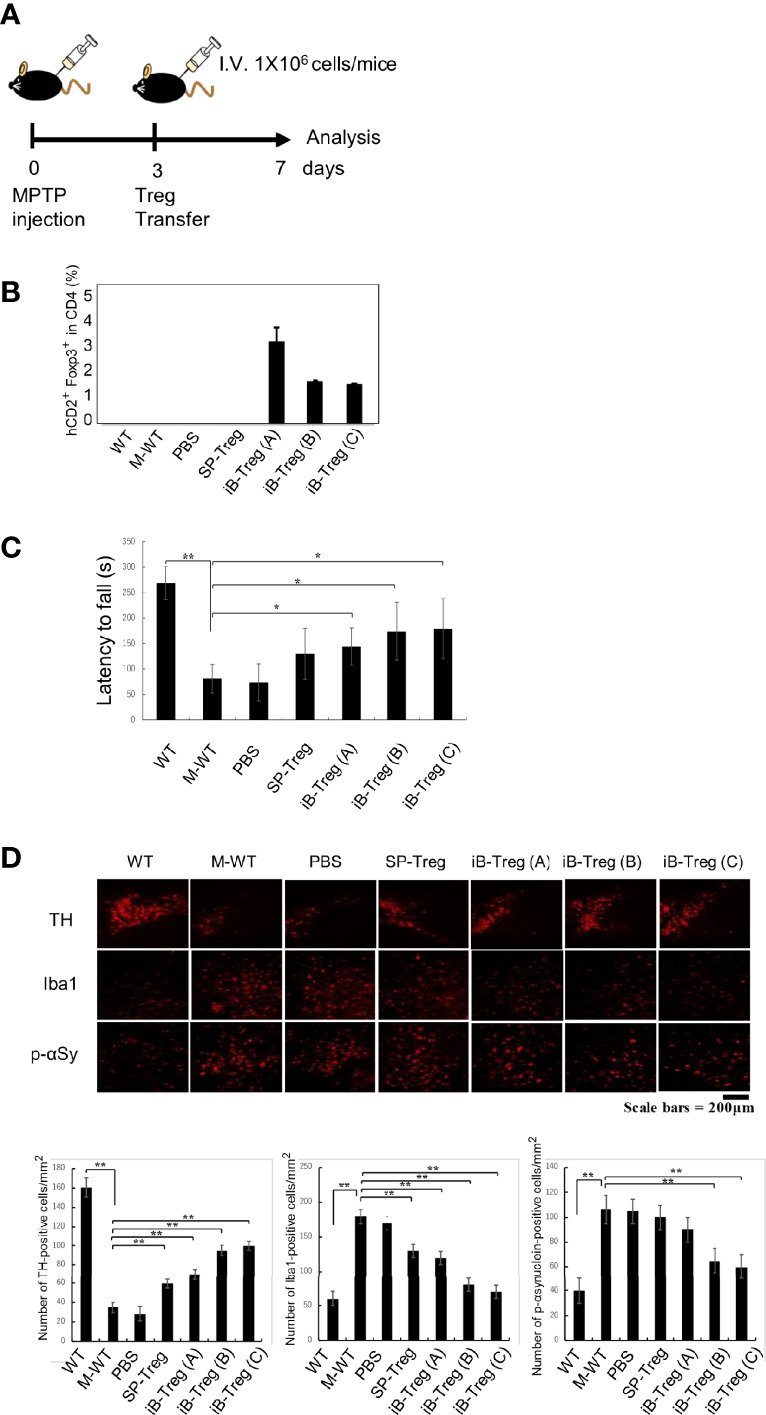
Therapeutic effects of iB-Tregs on a Parkinson’s disease model. **(A)** Parkinson’s disease model protocol. After 2 days of training, MPTP (20 mg/kg body weight) was administrated four times every 2 h and Tregs (1×10^6^) were injected intravenously on day 3 after MPTP treatment and analyzed on day 7. **(B–D)** MPTP-induced inflammation and injury of the substantia nigra. **(B)** Infiltrated T cells and Treg cells in the brain of MPTP-treated mice were analyzed using FACS, and the fraction of Foxp3^+^hCD2^+^ cells in the brain were estimated. **(C)** The rod test score for MPTP-treated mice in control, PBS-transfected (PBS), splenic Treg-transfected (SP-Treg), and iB-Treg-transfected **(A–C)** groups. **(D)** Dopaminergic neurons were stained with anti-TH antibody, activated microglia cells were stained with anti-Iba1(Iba1) antibody, and neural damage was stained with anti-p-αSynuclein antibody, and the images were quantified. * P<0.05 ** P<0.01.

Then, iB-Treg transfer was performed in wild-type mice on day 3 after MPTP administration (**Figure 5A**). Although we could not definitely conclude the brain infiltration efficiency due to very low number of infiltrated T cells, it was very clear that transferred Foxp3^+^hCD2^+^ iB-Tregs was accumulated in the brain more efficiently than splenic Tregs (**Figure 5B** and **Supplementary Figure 3H**). Next, we evaluated the motor coordination using the rotarod performance test for Parkinson-related symptoms 7 days after MPTP administration. The motor dysfunction induced by MPTP was restored by the transfer of Tregs (**Figure 5C**). This recovery of motor dysfunction was more pronounced in iB-Tregs compared with splenic Tregs, and it was also more pronounced in iB-Tregs (B) and iB-Tregs (C) than in iB-Tregs (A). Inhibition of dopaminergic neuronal degeneration and inflammation in the substantia nigra measured by microglial activation and phosphorylated α-synuclein expression were also more pronounced in B- and C-type iB-Tregs compared with splenic Tregs and iB-Tregs (A) (**Figure 5D**). These data strongly support our proposal that the astrocyte co-culture system, in the presence of IL-33 and serotonin, can generate genetical and functional brain Treg-like cells *in vitro*.

## Discussion

Tissue Tregs, which are naturally resident in various organs or accumulate in damaged tissues, have been extensively studied in recent years. It has been shown that tissue Tregs gradually acquire their characters in their lymph nodes and then become mature tissue Tregs within tissues (4–6, 23). However, environmental factors that are necessary for tissue Treg development remain to be elucidated. In this study, we attempted to generate tissue Tregs, especially brain Tregs, *in vitro*. To the best of our knowledge, no *in vitro* methods to induce tissue Tregs have been reported to date. Using a mouse cerebral infarction model, we showed that brain Tregs are activated and proliferate in both cervical lymph nodes and in the brain through the action of IL-2, IL-33, and serotonin (10). Brain Tregs have also been shown to interact with astrocytes and regulate astrogliosis through Areg. On the basis of this evidence, we hypothesized that Tregs mature into brain Tregs by receiving signals from the microenvironment of the brain tissue. We, therefore, co-cultured brain Tregs with brain cells such as astrocytes and microglia. At first, we thought that microglia, which also function as antigen-presenting cells expressing class II major histocompatibility complex (MHC), could stimulate Tregs that recognize antigens presented on class II-MHCs. However, Tregs did not proliferate in co-culture with microglia and many died. The reason that microglia cells can inhibit Treg proliferation is unknown, but we speculate that they may secrete growth inhibitory or cytotoxic factors such as IL-6 and kynureine (24). On the other hand, Tregs proliferated significantly and expressed activation markers of Tregs such as Foxp3 and CD25 at high levels when they were co-cultured with astrocytes. Astrocytes activated Tregs in an adhesion-dependent manner. Integrins and transforming growth factor β (TGF-β) are likely candidates as activators (25). Retinoic acid may be involved in Foxp3 expression, since retinaldehyde dehydrogenases are shown to be expressed in primary astrocytes (26). We found that some notch genes were expressed on Tregs and *Jag1* was expressed on astrocytes. Therefore, it is possible that Jag1 on astrocytes is involved in induction of Treg proliferation. In addition, several semaphorins, such as *Sema4a*, *Sema5b*, and *Sema6d*, were highly expressed on astrocytes, and on the other hand, semaphorin receptors, including Neuopillin-1 (*Nrp1*), which has been shown to be important for Treg survival (27), were highly expressed on Tregs. Thus, Semaphorin signaling may also be involved in an adhesion-dependent manner.

However, co-culture with astrocytes alone did not induce expression of tissue Treg signature genes such as GATA3, PPARγ, KLRG1, and ST2. Therefore, we added various cytokines and bioactive substances to the co-culture system and showed that IL-4 was the most potent inducer of GATA3, PPARγ, and ST2. However, IL-4 strongly inhibited Treg proliferation in the co-culture system and did not induce KLRG1 expression. We are skeptical that IL-4 is actually involved in the development of brain Tregs because IL-4 expression was barely detectable in the brain *in vivo* in our stroke experiments. On the other hand, IL-33 is a reasonable candidate because IL-33 is known to be involved in proliferation and maturation of almost all tissue Tregs. We also found that serotonin alone induces Htr7 expression, which is characteristic of brain Tregs but not in other tissue Tregs (10). Although the molecular mechanism is currently unknown, Htr7 signals may have a positive feedback induction mechanism. Further study is needed to clarify the complex mechanism of brain Treg induction *in vitro*.

iB-Tregs that were generated using our method showed a gene expression pattern that was similar, but not identical, to that of brain Tregs. The expression of CCR6, which is important for brain infiltration, was somewhat decreased, and the expression of Areg, which is an important tissue repair factor, was also low. It is likely that the iB-Tregs generated using the current method are early brain Tregs that are similar to those generated in cervical lymph nodes. Thus, further development and maturation in the brain may be necessary to become mature brain Tregs. Identification of maturation factors in the brain is important for the future generation of complete brain Tregs *in vitro*. Another important issue is that the TCR specificity of iB-Tregs. iB-Tregs expanded by anti-CD3 antibodies may not specifically recognize brain antigens. This may be a reason why the infiltration efficiency into the brain was lower than expected when iB-Tregs were transferred into brain disease models. To obtain complete brain Tregs in the future, it is important to identify and isolate TCRs that are specific for brain antigens and introduce them into iB-Tregs.

We expected that iB-Tregs (C) work better than iB-Tregs (B) due to higher chemokine receptor genes expression. However, *Htr7* and *Ccr6* expression is rather lower in iB-Tregs (C) than in iB-Tregs (B). We have shown that deletion of *Htr7* and *Ccr6* in Tregs markedly reduced their ability to infiltrate and proliferate in the brain after cerebral infarction (10). Therefore, iB-Tregs (B) are closer to brain Tregs present in ischemic brain. On the other hand, iB-Tregs (C) seem to ameliorate Parkinson’s disease model more efficiently than iB-Treg (B), although serotonin is considered to be therapeutic to Parkinson’s disease. We notice that *Penk* expression is higher in iB-Tregs (C) than in iB-Treg (B). *Penk* overexpression in neurons has been shown to ameliorate Parkinson’s disease (28). Therefore, it is ideal to generate iB-Tregs expressing functional genes present in both iB-Tregs (B) and (C) to ameliorate symptoms in Parkinson’s disease.

Despite such limitations, a method to generate a large number of brain-specific Tregs *in vitro* may be important for developing new therapies against brain inflammatory diseases. For example, naïve Treg transfer or Treg upregulation by IL-2/IL-2 antibody complexes have been reported to alleviate symptoms of cerebral infarction models (11, 29). If we can use iB-Treg transfer for brain infarction, the therapeutic effects may be higher than those of naïve Tregs. Tregs were reported to play important regulatory roles in the neurological symptoms of neurological diseases. In this study, we demonstrated that iB-Treg transfer more effectively ameliorates Parkinson’s disease than conventional Tregs. Brain Tregs may also be therapeutic for various cerebrospinal diseases such as spinal cord injury, multiple sclerosis, and neurodegenerative diseases including as Alzheimer’s disease. Using our experimental system, it is difficult to distinguish whether iB-Tregs function inside the brain or in the secondary lymphoid organs. For clinical application, it is necessary to consider gene expression differences in tissue Tregs between mice and humans. For example, human tissue Tregs in several organs do not express ST2 (30). Considering such differences in gene expression of tissue Tregs, it is extremely important to examine cytokines and other stimuli for human iB-Treg generation. Nevertheless, our study proposes that induction of mature brain-specific Tregs may be a novel therapeutic approach to alleviate symptoms in stroke and other CNS diseases.

## Materials and Methods

### Mice

C57BL/6J mice were purchased from Tokyo Laboratory Animals Science Co., Ltd. (Tokyo, Japan). *Foxp3-hCD2 and Cd3ϵ^−/−^* mice were described previously (10, 31, 32). Mice, aged 8–12 weeks and weighing 20–30 g were used under co-housing conditions in specific pathogen-free facilities. Animal experiments were performed in strict accordance with the recommendations in the Guidelines for Proper Conduct of Animal Experiments of the Science Council of Japan. All experiments were approved by the Animal Research Committee and Ethics Committee of Keio University and Kyushu University.

### Primary Neonatal Mouse Brain Cultures

Primary mouse glial cells were prepared as previously described (15–17). Briefly, neonatal mouse brains were excised and the meninges were removed. The isolated brains were minced, treated with trypsin and DNase I, and the cell fraction was passed through a 100 µm cell strainer followed by seeding into poly-L-lysine-coated T75 flasks. Cells were cultured in a CO_2_ incubator for overnight in DEME medium containing 10% FCS supplemented with 4 mM L-glutamine. After 10–20 days, the microglia fraction was collected by gently rotating the flasks and floating cells were seeded (2×10^6^ cells) into T25 flasks. After incubation for 3 h in a CO_2_ incubator, attached cells were washed with serum-free DMEM medium and further cultured for overnight in the serum free medium. Astrocytes were cultured in an air shaker at 37°C for 1 day with shaking (130 times/min), washed with PBS, and further cultured in a CO_2_ incubator for 24 h with DEME medium containing 10% FCS supplemented with 4 mM L-glutamine. The cells were then trypsinized, passed through a cell strainer (100 µm), seeded (2×10^6^ cells) into T25 flasks, and cultured in a CO_2_ incubator for 1 week. Astrocytes accounted for more than 90% of the total.

### Co-Culture With Tregs

Tregs were isolated from the spleen and lymph nodes of *Foxp3-hCD2* knock-in mice as described previously (9). hCD2^+^ T cells were isolated using EasySep Mouse APC-Positive Selection kit (VERITAS) using FITC-conjugated anti-mouse CD4 monoclonal antibody (mAb) (RM4-5; eBioscience™), APC-conjugated anti-hCD2 mAb (RPA-2.10; eBioscience™), and Fixable Viability Dye eFluor™ 780 (eBioscience™) to exclude dead cells. Tregs were further sorted using a cell sorter (SH800; SONY Biotechnology). Tregs and glial cells were co-cultured using the method described below. The culture medium was removed from the semi-confluent glial cells culture, and the Treg cells (1.0 × 10^6^/well) were then added and cultured in the presence of 2-mercaptoethanol (×1000 dilution), anti-CD3 antibody (2 μg/mL), anti-CD28 antibody (2 μg/mL), and IL-2 (20 ng/mL) for 3–6 days. Half of the medium was changed every 2 days. Anti-CD3 and anti-CD28 antibodies were added only on the first day. Various cytokines were added at the indicated concentrations described below. To prepare the CM, semi-confluent astrocytes or microglia (2×10^5^/well) in 24 well dishes were washed three times using serum-free medium, cultured in DEME medium containing 10% FCS supplemented with 4 mM L-glutamine for 24 h, collected in sterile tubes, and stored at −25°C until use. CM (0.5 mL/well) was added to the 0.5 mL Treg culture in 24-well dishes.

All cytokines were purchased from Peprotech, and CCL20 and CCL1 were purchased from BioLegend. Final concentrations were indicated as follows: IL-2 (20 ng/mL), IL1-β (20 ng/mL), IL-4 (20 ng/mL), IL-6 (20 ng/mL), IL-10 (20 ng/mL), IL-33 (20 ng/mL), tumor necrosis factor α (20 ng/mL), EGF (20 ng/mL), TSLP (20 ng/mL), macrophage–colony-stimulating factor (20 ng/mL), BDNF (20 ng/mL), Areg (20 ng/mL), CCL20 (20 ng/mL), and CCL1 (20 ng/mL). All the following chemicals were from Sigma-Aldrich: prostaglandin E2 (1 μM), pioglitazone hydrochloride (1 μg/mL), LPS (500 ng/mL), Vitamin A (25 nM), Vitamin C (10 μg/mL), and serotonin hydrochloride (10 μM).

### FACS and Quantitative PCR Analysis

Co-cultured cells in flasks were collected with ethylenediaminetetraacetic acid, centrifuged, and resuspended with MACS buffer. After treatment with Fc blockers (anti-CD16/32 antibody) at 4°C for 30 min, cells were washed and stained with the indicated antibodies at 4°C for 30 min for FACS analysis. For qPCR, hCD2^+^ T cells were isolated using auto-MACS (Miltenry) and analyzed using real time (RT)-qPCR (Bio-Rad), as reported previously (9, 33). For total cell RNA sequencing (RNAseq) analysis, hCD2^+^ T cells were also isolated using the auto-MACS, and total RNA was purified using a MagMAX mirVana Total RNA Isolation Kit (Thermo Fisher).

The following antibodies for FACS were purchased from eBioscience™: rat BV480- conjugated anti-mouse CD196 (CCR6), rat BV480- conjugated IgG2a, κ isotype control, PerCP-eFluor 710-labeled anti-IL-33R (ST2) mAb (RMST2-33), rat PerCP-eFluor 710-labeled IgG2b kappa isotype control (eB149/10H5), PE-Cyanine7-labeled anti-KLRG1 mAb (2F1), PE-Cyanine7-labeled Syrian hamster IgG isotype control, rat biotin-conjugated anti-mouse CD198 (CCR8) mAb, rat biotin-conjugated κ isotype control antibody, PerCP-eFluor™ 710 conjugated streptavidin, APC-conjugated anti-CD4 mAb (RM4-5), APC, rat BV480- conjugated anti-mouse CD19 mAb, rat BB515-labeled anti-CD11b mAb, PE-conjugated anti-Foxp3 mAb, PE-conjugated anti-CD25 (PC61.5) mAb, rat BB700-labeled anti-mouse CD8a mAb, and PE-Cyanine7-conjugated CD45 mAb (30-F11).

### Parkinson’s Disease and Other Brain Disease Models

A 1-methyl-4-phenyl-1,2,3,6-tetrahydropyridine (MPTP) solution (100 µL) was injected intraperitoneally at 20 mg/kg body weight four times every 2 h, as described in a previous report (12). Three days after the last injection, Tregs were adoptively transferred into MPTP-treated recipient mice (n=5–7 mice). Seven days after MPTP injection, mice were sacrificed and the brains were processed for immunohistochemical analysis. To assess the effect of MPTP on behavior, the rotarod test was performed to measure motor coordination (34). The acceleration mode (2–16 rpm for at least 2 min) and constant velocity mode (16 rpm for 5 min) were performed as training periods for 2 days before MPTP administration. In the real rotarod test, the rod was rotated in the constant velocity mode (16 rpm) and the time until the mice fell from the rod was measured. EAE and cerebral infarction models were performed as previously described (35, 36).

### Mouse Model of Ischemic Stroke

Male mice, aged 8–12 weeks and weighing 20–30 g, were used for focal brain ischemia experiments (10). There was no significant difference in weight or age between wild-type mice and any of the knockout groups. We used a transient MCAO model that was constructed using an intraluminal suture. The method of inducing this transient suture MCAO model was previously described (10). The mice were anaesthetized with isoflurane in a mixture of 70% nitrous oxide and 30% oxygen. During the MCAO procedure, the head temperature was kept at 36°C using a heat lamp. Sixty minutes after MCAO, the brain was re-perfused by removing the intraluminal suture.

### Immunohistochemical Staining of the Brain

Frozen midbrain sections (20 µm thick) were prepared using a cryostat and stored at −20°C until staining. After air-drying for 20 min, sections were fixed with 3.7% formalin for 5 min and treated with 0.1% TritonX-100 for 5 min. The sections were blocked for 50 min at room temperature using blocking solution (0.2% skim milk). The first antibodies were anti-TH antibody (GeneTex, GTX113016), rabbit anti-Iba1 antibody (Wako; 019-19741), and anti-phosphorylated α-synuclein antibody (pSer129) (GeneTex, GTX54991), and they were diluted in blocking solution and then treated with the tissue sections overnight at 4°C. The next day, the sections were washed with PBS and reacted with the fluorescently labeled second antibodies diluted in blocking solution and incubated overnight at 4°C. After washing with PBS, the sections were sealed with an encapsulant and observed and photographed with a BZ-X700 fluorescence microscope (Keyence). Quantification of striatal TH was performed using BZ-H4A quantification software.

### FACS Analysis of T Cells from the Brain and Spinal Cord

Brains and spinal cords were removed from mice, incubated with collagenase D and DNase I in a shaker (200 rpm) for 30 min at 37°C, and then centrifuged at 2000 rpm for 20 min in 30%/70% Percoll. After centrifugation, the intermediate layer was collected and resuspended in MACS Buffer. FACS analysis was then performed as described (10).

### RNA Sequencing

Library preparation was used NEBNext PolyA stranded Ultra II Directional RNA Library Prep (New England BioLabs, MA) by Azenta. The libraries were sequenced on Illumina HiSeq 1500 sequencers (Illumina, San Diego, CA). Sequence reads in FASTQ format was applied to FastQC (v0.11.4) to assess sequence quality. For adapter trimming, Trim Galore! (v0.6.0) was used. The sequence reads (SP-Treg, Ischemia-Treg, iB-Treg (A), iB-Treg (B), iB-Treg (C), NC, EAE.Br and EAE.SC) were mapped to mouse reference genome GRCm38/mm10 from Ensembl (release 102) using HISAT2 (version 2.2.1). The mapped reads were counted for genes using featureCounts (v.2.0.0). These data were further normalized using the DESeq2 R package (v.1.30.1) and an rlog (regularized logarithm) transformation was applied (37). PCA was performed on these data. The SP-Treg, Ischemia-Treg, iB-Treg (A), iB-Treg (B) and iB-Treg (C) data were extracted from the transformed data and heat maps were generated using the ggplot2 R package (v.3.1.1).

### Statistical Analysis

The results are expressed as the mean ± SD from three to five independent experiments. The Student’s *t*-test was used to evaluate whether there was a significant difference between the two groups. In all cases, P values less than 0.05 were considered significant (* P<0.05, ** P<0.01).

## Data Availability Statement

The datasets presented in this study can be found in online repositories. The names of the repository/repositories and accession number(s) can be found below: Gene Expression Omnibus (GEO)under accession number GSE203050.

## Ethics Statement

The animal study was reviewed and approved by the Animal Research Committee and Ethics Committee of Keio University and Kyushu University.

## Author Contributions

SY, MI, and AY designed the research project and performed experiments; AM, MO, YH, and MI-K helped with experiments; CK and MS performed the data analyses; and MI and AY wrote the manuscript with input from all authors. All authors contributed to the article and approved the submitted version.

## Funding

This work was supported by JSPS KAKENHI 21H05044, 19H04817, 21K19382, 21H02719, 22K19444, 21H00432, AMED-CREST 22gm1110009, MOON SHOT JP22zf0127003h, AMED-PRIME 22gm6210012, AMED- 22wm0425011 the Yasuda Medical Foundation, Research grant from the Chemo-Sero-Therapeutic Research Institute, the Kishimoto Family Foundation, the Tomizawa Jun-ichi & Keiko Fund of Molecular Biology Society of Japan for Young Scientist, the Mitsubishi Foundation, the Mochida Memorial Foundation for Medical and Pharmaceutical Research, the Takeda Science Foundation, the Uehara Memorial Foundation, the Naito Foundation, the Kanae Foundation, the SENSHIN Medical Research Foundation, the Astellas Foundation for Research on Metabolic Disorders, the Novartis Research Grant, the Nakajima Foundation, the Kinoshita Foundation, the Inoue Research Award for Young Scientists, a Life Science Research Award, and Keio Gijuku Academic Developmental Funds.

## Conflict of Interest

The authors declare that the research was conducted in the absence of any commercial or financial relationships that could be construed as a potential conflict of interest.

## Publisher’s Note

All claims expressed in this article are solely those of the authors and do not necessarily represent those of their affiliated organizations, or those of the publisher, the editors and the reviewers. Any product that may be evaluated in this article, or claim that may be made by its manufacturer, is not guaranteed or endorsed by the publisher.

## Supplementary Material

Supplementary Figure 1 Effect of conditioned medium from astrocyte and microglia on Treg activation and ST2 expression. **(A, B)** Tregs were cultured in the presence or absence of AMC (conditioned medium from astrocytes) or MCM (conditioned medium from microglia) for 3 days in the presence of IL-2 and IL-4. Foxp3, CD25, and ST expression in Tregs was analyzed using FACS. **(C, D)** Tregs were co-cultured with astrocytes in the presence or absence of MCM for 3 days in the presence of IL-2 and IL-4. Foxp3, CD25, and ST2 expression in Tregs was analyzed using FACS.

Supplementary Figure 2 Therapeutic effects of iB-Tregs in an EAE model. Tregs (1×10^6^) were injected intravenously on day 16 after EAE onset and analyzed until day 21 **(A)**. Clinical scores for EAE in control, PBS-transfected (PBS), SP-Treg-transfected (SP-Treg), and iB-Treg-transfected **(A–C)** mice are shown in **(B)**. * P<0.05

Supplementary Figure 3 Establishment of a Parkinson’s disease model by MPTP administration. **(A, B)** Rotarod test protocol. After 2 days of training, MPTP (20 mg/kg body weight) was administration four times every 2 h, and analysis took place on day 7. The rod was rotated in a constant velocity mode (16 rpm), and the time (s) until the mice fell from the rod was measured (latency to fall). **(C–E)** The effect of X-ray irradiation on MPTP-induced inflammation and injury of the substantia nigra **(C)**. Dopaminergic neurons were stained with anti-TH antibody, activated microglia cells were stained with anti-Iba1 antibody, and neural damage was stained with anti-p-αSynuclein antibody **(D)**, and the images were quantified in **(E)**. T cell infiltration into the brain of MPTP-treated mice was analyzed using FACS **(F–H)** Infiltrated T cells and Treg cells in the brain of MPTP-treated mice were analyzed using FACS. * P<0.05 ** P<0.01
