# Supplementary figures and images for: In Vitro Generation of Brain Regulatory T Cells by Co-culturing With Astrocytes
Source: Front Immunol. 2022 Jul 15;13:960036. doi: 10.3389/fimmu.2022.960036 (PMC9335882; doi:10.3389/fimmu.2022.960036)

A

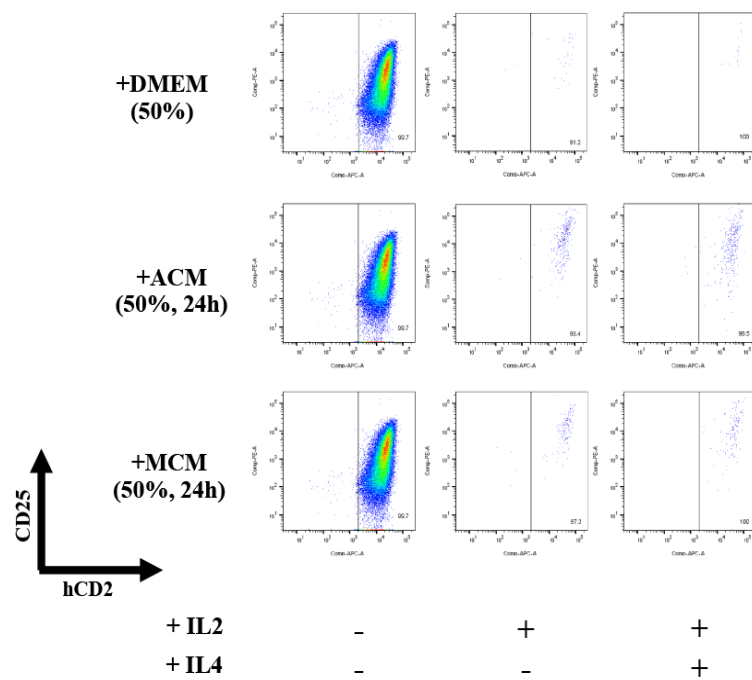

B

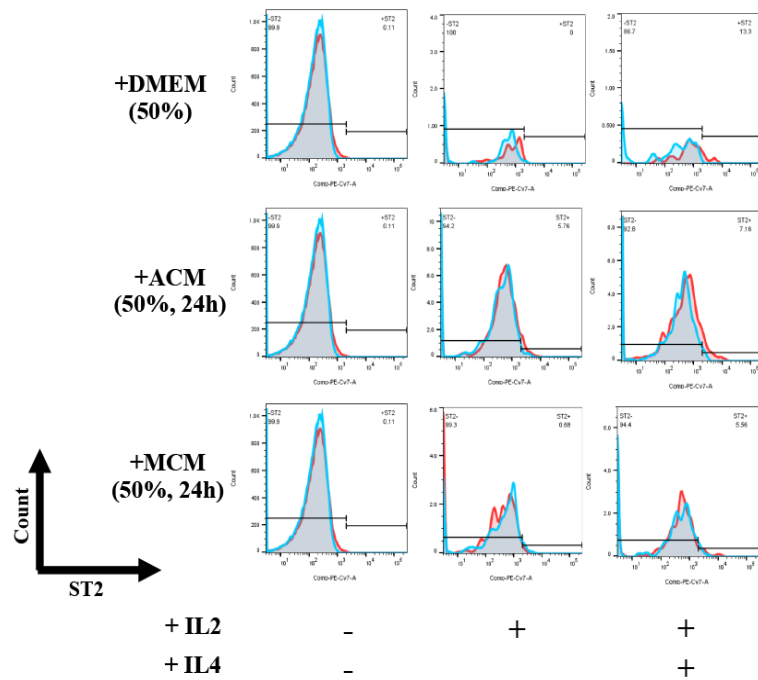

C

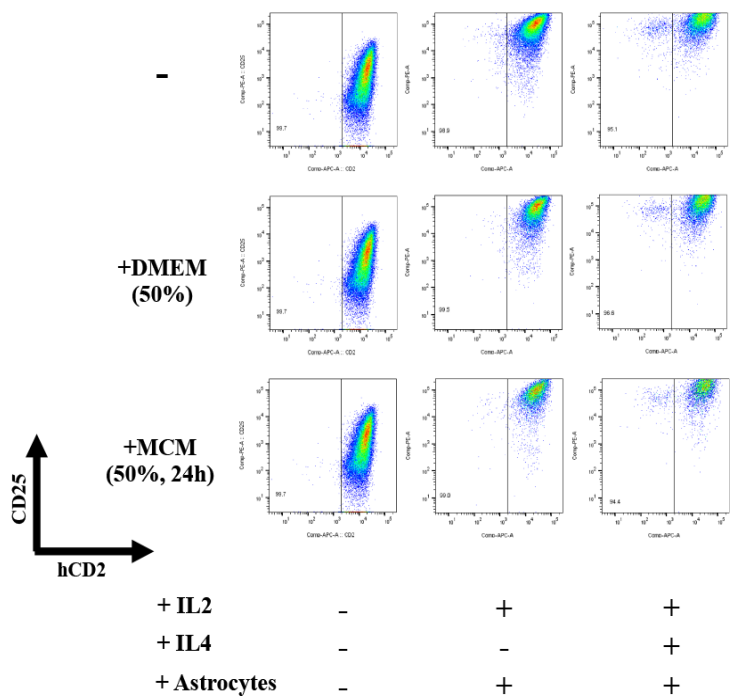

D

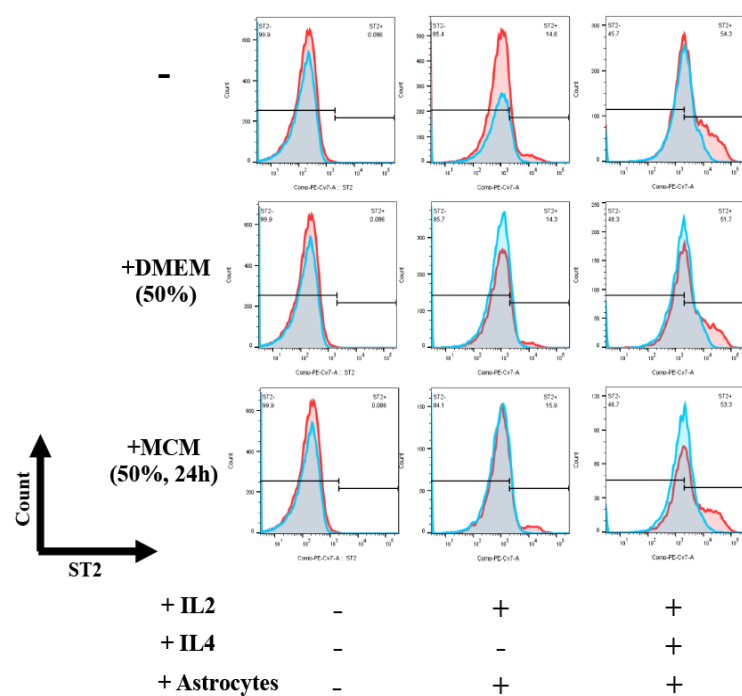

Supplementary Fig.1

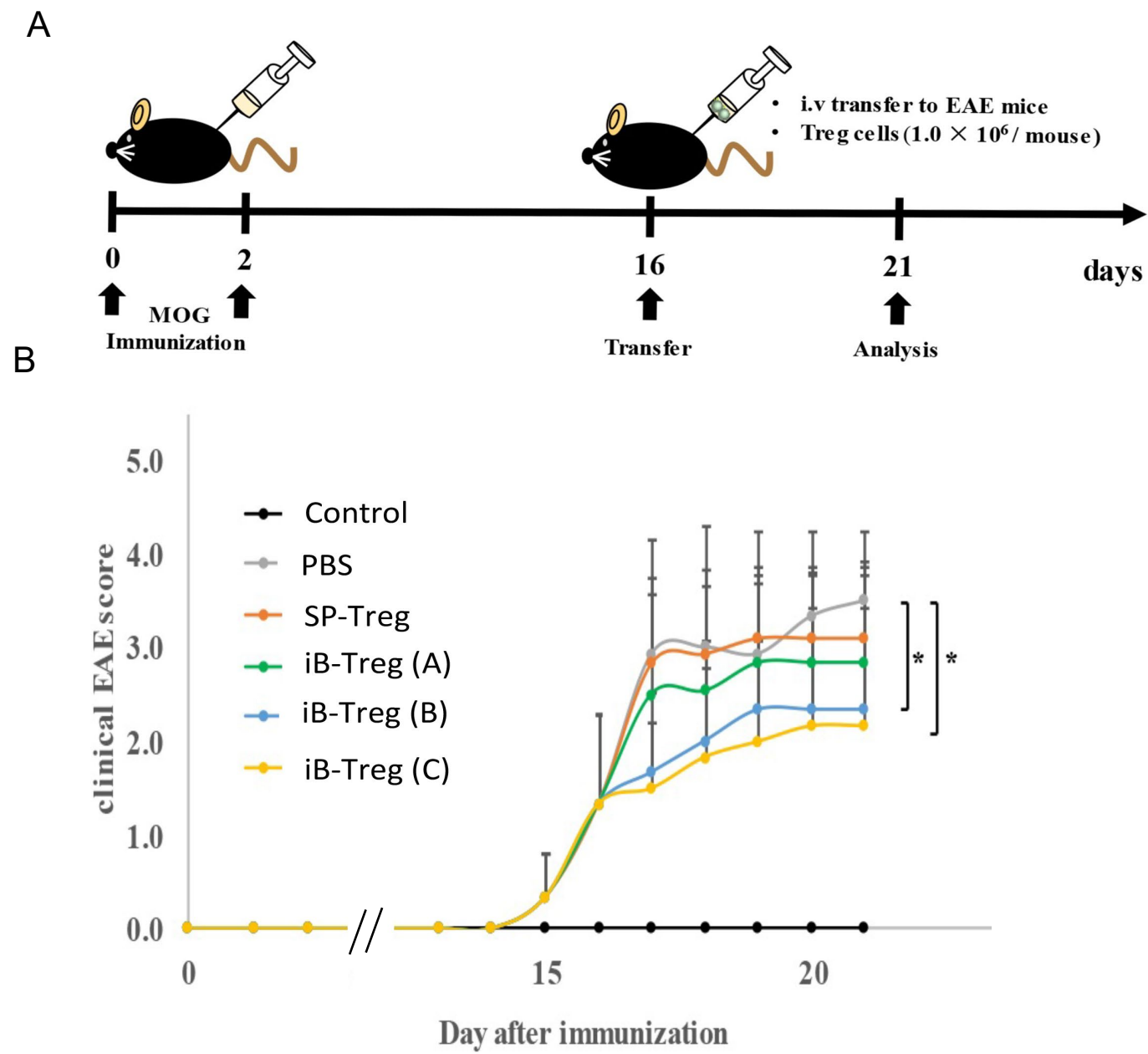

Supplementary Fig.2

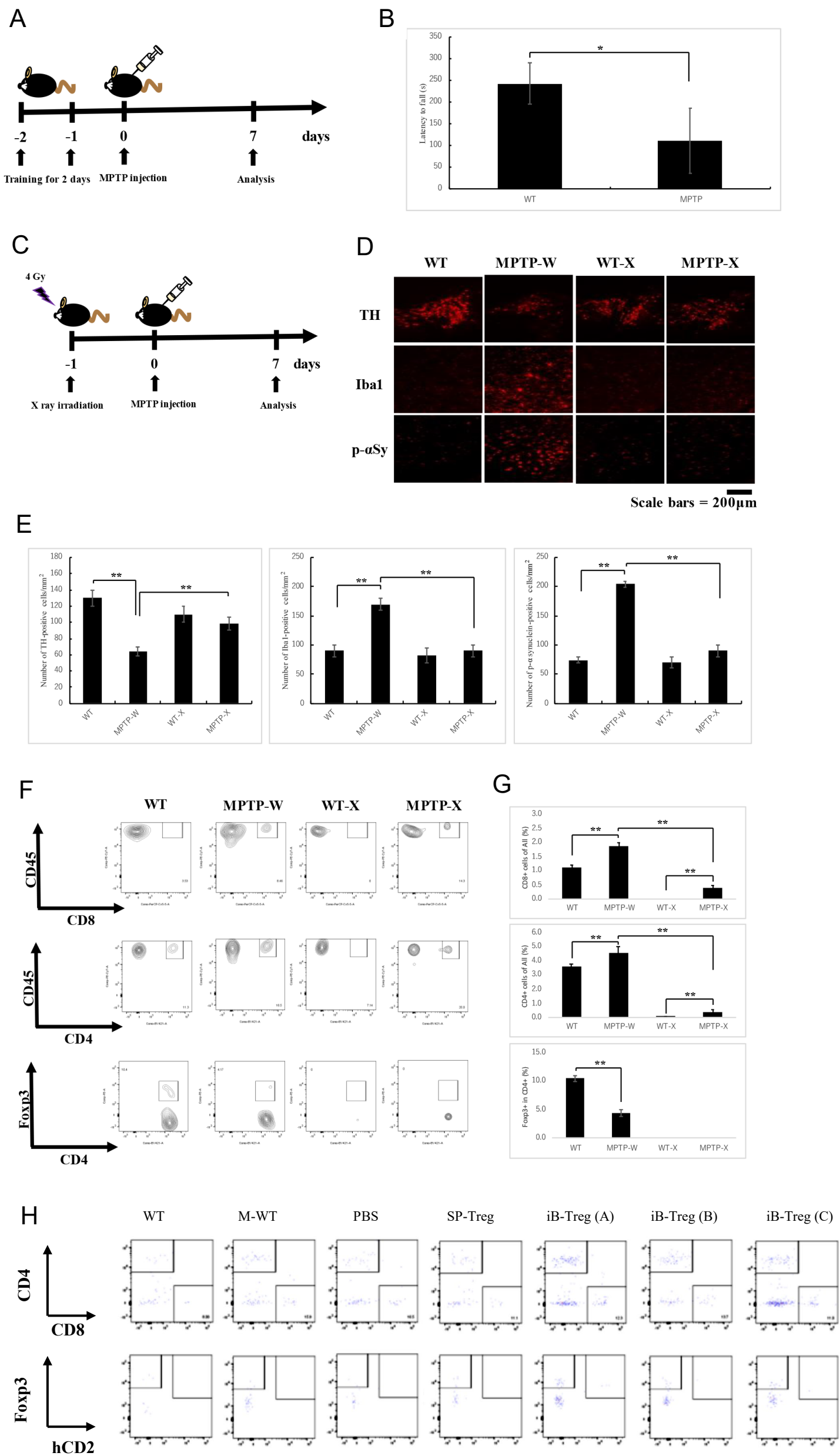

Supplementary Fig. 3

Supplement: Supplementary Figure 1 — Effect of conditioned medium from astrocyte and microglia on Treg activation and ST2 expression. (A, B) Tregs were cultured in the presence or absence of AMC (conditioned medium from astrocytes) or MCM (conditioned medium from microglia) for 3 days in the presence of IL-2 and IL-4. Foxp3, CD25, and ST expression in Tregs was analyzed using FACS. (C, D) Tregs were co-cultured with astrocytes in the presence or absence of MCM for 3 days in the presence of IL-2 and IL-4. Foxp3, CD25, and ST2 expression in Tregs was analyzed using FACS. [file Image_1.pdf]
